# Post-translational protein modifications in schizophrenia

**DOI:** 10.1038/s41537-020-0093-9

**Published:** 2020-03-02

**Authors:** Toni M. Mueller, James H. Meador-Woodruff

**Affiliations:** Department of Psychiatry and Behavioral Neurobiology, University of Alabama at Birmingham, Birmingham, AL 35233 USA

**Keywords:** Schizophrenia, Molecular neuroscience

## Abstract

Research investigating the pathophysiology of schizophrenia has not yet precisely defined the molecular phenotype of this disorder. Many studies have investigated cellular dysfunction by examining expression levels of molecular targets in postmortem patient brain; however, inconsistencies between transcript and protein measures in schizophrenia are common in the field and represent a challenge to the identification of a unified model of schizophrenia pathogenesis. In humans, >4800 unique proteins are expressed, and the majority of these are modified by glycans and/or lipids. Estimates indicate ~70% of all eukaryotic proteins are modified by at least one type of glycosylation, while nearly 20% of all proteins are known to be lipid-modified. Protein post-translational modification (PTM) by glycosylation and lipidation rely on the spatiotemporal colocalization of enzyme, substrate, and glycan or lipid donor molecule and do not require an upstream “blueprint” or specialized processing machinery for synthesis. Glycan and lipid PTMs can thus facilitate cellular adaptation to environmental signals more rapidly than changes of gene or protein expression, and can significantly impact the localization, function, and interactions of modified substrates, though relatively few studies in schizophrenia have evaluated the PTM status of target proteins. A growing body of literature reports glycosylation and lipidation abnormalities in schizophrenia brain as well as in patient peripheral fluids. In this review, we explain the functional significance of key glycan and lipid PTMs and summarize current findings associated with abnormal glycosylation and lipidation in this illness.

## Introduction

Schizophrenia is a complex neuropsychiatric disorder characterized by dysregulated molecular processes in multiple brain regions, cell types, and neurotransmitter systems.^1–3^ Despite decades of research, advancements in treatment and identification of novel therapeutic targets in schizophrenia have been infrequent.^4^ Previous research in schizophrenia has primarily focused on identifying molecular abnormalities related to specific neurotransmitter systems and neuronal subpopulations, while conceptualization of neuropsychiatric illness as a broader defect of cell biology has not been as widely considered. Increasing evidence of defects in schizophrenia that persist across all cell types, such as mitochondrial dysfunction and cellular metabolic abnormalities, suggests that the disorder may arise from a central molecular abnormality which differentially impacts function in a cell-specific manner. Alterations to activity-dependent central cell signaling processes, such as the expression and regulation of protein post-translational modifications (PTMs) may represent a common mechanism underlying cellular dysfunction in the disorder that could explain variable disruptions in multiple cell-types, brain regions, and neurotransmitter systems.

Recent reports on postmortem brain research in schizophrenia have suggested protein PTMs as a potential target of investigation.^5,6^ Altered glycan and lipid PTMs (glycosylation and lipidation, respectively), which integrate both genetic and environmental influences on central nervous system development and function, could contribute to variability of symptom expression and patient treatment response in this multifactorial neuropsychiatric illness. Ongoing research into PTM processing mechanisms, PTM status of specific substrates, and investigation of PTMs that influence multiple functional pathways may also serve to reconcile inconsistent genetic, transcriptomic, and proteomic findings previously reported in the literature.

In this review, we describe the functional importance of glycan and lipid protein PTMs and provide an overview of current evidence in schizophrenia of defects of protein glycosylation and lipidation. These processes affect cellular functions downstream of transcription and protein translation and have not yet been extensively characterized in this disorder. We examine current evidence for altered PTM processing and expression in schizophrenia, including reports of abnormal total levels of specific PTM structures, evidence of neurotransmission-associated proteins with altered PTMs, and dysregulation of enzymes which create, modify, or degrade PTM structures. We also synthesize PTM-associated findings in schizophrenia with existing basic science research on PTMs and PTM processing and function that are related to pathways and signaling molecules with known relevance to schizophrenia pathophysiology. The glycan and lipid PTMs we review include *N-*linked and *O*-linked glycosylation, prenylation, *N*-myristoylation, and *S*-palmitoylation, as well as relevant substrate-specific PTMs.

Many studies in schizophrenia have investigated molecular correlates of neuropsychiatric illness in the context of DNA (e.g. single nucleotide polymorphism (SNP) expression, genome-wide association studies (GWAS)), RNA (e.g. transcriptomics, in situ hybridization studies), and protein (e.g. expression levels, localization, interaction networks, and functional pathways), but the results of these studies are often inconsistent. For example, in postmortem schizophrenia brain research it is not uncommon to find that reported mRNA expression level changes do not necessarily correlate with changes in protein expression.^7–9^ Investigations of individual proteins or protein families rarely consider the potential impact of target protein glyco- or lipoforms, even in cases where it has been established that the PTM state of the molecule can significantly influence molecular interactions and/or substrate function. This does not discount the relevance of gene and protein level assessments to identify molecular pathways and functional processes perturbed in the illness; however, consideration of substrate-specific modulators of function such as PTM status evaluated in conjunction with gene and protein measures can provide researchers a more comprehensive indication of target function. Because PTM processing does not require an upstream “blueprint” like transcription or translation, and instead only requires the appropriate colocalization of enzyme, substrate, and modifying biomolecule, PTMs are dynamically regulated more rapidly than alterations to gene and protein expression, and many cellular functions regulated by the PTM status of key proteins are sensitive to spatiotemporal and metabolic aspects of the cellular environment. This review defines the processing mechanisms and functional significance of key glycan and lipid PTM subtypes, and summarizes the current evidence for dysregulated glycosylation and lipidation processes in schizophrenia.

## Glycan PTMs

**Fig. 1:**
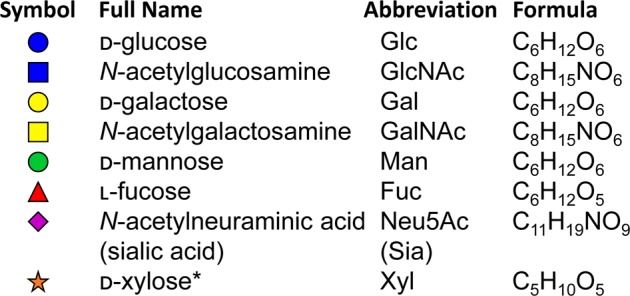
Common monosaccharides in human glycans. Symbols and abbreviations are reported following the standards established by the Symbol Nomenclature for Glycans (SNFG) Discussion Group in conjunction with NCBI and PubChem.^163^ Information in parenthesis represents alternate nomenclature commonly referenced in scientific reports published prior to the widespread acceptance of a uniform naming convention. *d-xylose is not synthesized by humans, but xylose obtained from dietary sources is incorporated into human glycans.

Glycosylation is an enzyme-mediated process whereby a carbohydrate or carbohydrate structure (also referred to as a glycan) is attached to a protein, lipid, or glycan substrate. The most common protein glycosylation pathways in the cell include *N*-linked and *O-*linked glycosylation, which indicate whether a glycan is attached to the substrate amino acid via a nitrogen (*N*-linked/*N*-glycosylation) or oxygen (*O-*linked/*O*-glycosylation) molecule. Monosaccharides most commonly incorporated into mammalian glycans, and their commonly accepted graphic symbols and nomenclature, are summarized in Fig. 1.

Nearly 2% of the human genome encodes glycosyltransferases, glycosidases, or other glycan-modifying enzymes,^10^ suggesting the importance of glycosylation pathway activity in cell biology. Protein levels of glycosylation-associated enzymes are dynamically regulated during development, and glycan structures can be influenced by the cell type or tissue in which substrates are expressed. Previous reviews^10–12^ provide concise summaries of the many and varied roles that glycans play in biological systems, while “Essentials of Glycobiology” (www.ncbi.nlm.nih.gov/books/NBK310274) offers detailed information about the chemistry, enzymology, substrates, functions, and molecules associated with specific glycan structures.^13^

## Lipid PTMs

Protein lipidation is a PTM where a lipid molecule is covalently attached to a protein, most often enzymatically. There exist several types of lipidation, and the most extensively studied include modification by the enzymatic attachment of a fatty acid or isoprenoid molecule.^14–16^ Common forms of lipidation include prenylation (farnesylation and/or geranylgeranylation), *N-*myristoylation, and *S*-palmitoylation. Three less common substrate-specific lipidation subtypes of interest in schizophrenia research are *N*-palmitoylation and cholesterylation of Hedgehog (Hh) proteins,^15–18^ and octanoylation of ghrelin.^15,16,19^ Comprehensive reviews of multiple types of protein lipidation, including detailed information about the enzymology, substrates, and biochemistry of fatty acid and isoprenoid lipidation of proteins, as well as some other less common lipid PTMs, have been recently published.^14–18^

The addition of a lipid moiety promotes substrate affinity for organelle and cellular membranes by increasing protein hydrophobicity, and specific lipid modifications or combinations of lipidation can mediate the preferential localization of a substrate to a specific compartment or membrane domain.^16,20,21^ Lipid PTMs regulate cellular function by modulating protein–membrane interactions and substrate localization to ensure proper spatiotemporal distribution of modified proteins.^14,20,21^ Most of the “common” features of lipid PTMs and lipid PTM processing are more aptly described by highlighting the exceptions rather than defining a set of uniform rules amongst lipidation subtypes. For example, aside from cholesterol esterification and glycolipid attachment, protein lipidation occurs on the nucleophilic side chains of cysteine (Cys), serine (Ser), or lysine (Lys) residues or by attachment to the *N*-terminal NH_2_ group of an amino acid chain.^14–16^ *S*-palmitoylation, another exception, represents a uniquely reversible lipid modification that regulates the localization and activity of substrate molecules by cycles of de- and re-palmitoylation.^14–16,20,21^ Other lipid attachments, including *N*-palmitoylation, are stable and irreversible until protein degradation occurs in the lysosome.^15–17^

## Evidence for altered glycosylation and lipidation in schizophrenia

A majority of transmembrane and secreted proteins require proper glycosylation, and many membrane-associated proteins require correct lipidation to be effectively targeted and trafficked to their sites of functional activity. Abnormal PTM processing in schizophrenia has been identified in patient-derived samples including blood serum, cerebrospinal fluid (CSF), urine, and postmortem brain tissue, and dysregulated glycosylation and lipidation may represent pathways important to the onset and progression of schizophrenia symptoms. Abnormalities related to protein glycosylation which have been reported in schizophrenia or in association with antipsychotic administration are summarized in Tables 1 and 2, while lipidation-associated alterations are summarized in Table 3. Gene expression patterns of patients on typical antipsychotic medications in the early (≤4 years), intermediate (7–18 years), and late (≥28 years) stages of schizophrenia have been assessed by GWAS.^22^ Researchers used multiple methods of pathway and gene function analysis to identify dysregulated molecular pathways in the central nervous system over the course of illness progression. Gene ontology term enrichment analysis found biopolymer glycosylation, protein amino acid glycosylation, and glycoprotein biosynthesis enriched in intermediate stage patients. Pathway analysis of differentially expressed genes found that carbohydrate metabolism was among the top interaction networks identified in short-term illness, while lipid metabolism was identified in intermediate term illness. Analyses of the top differentially expressed genes from each stage of the disorder identified glycosylation/glycoprotein as a common dysregulated function between intermediate and long-term illness stages; while lipid metabolism, protein localization, and protein transport/modification were common among all illness stages.^22^ A more recent GWAS study in postmortem schizophrenia liver found differences in the gene expression patterns between patients on atypical versus typical antipsychotics for genes associated with cellular lipid metabolism, lipid biosynthesis, and lipid metabolism.^23^ Between patients and non-psychiatrically ill subjects differences in the expression of genes associated with response to stress and protein binding were identified, while genes associated with the endoplasmic reticulum (ER), ER-Golgi transport, and Golgi transport were reported for patients on typical antipsychotics. A recurring theme of these GWAS study results is that functions associated with protein targeting, trafficking, and localization manifest at all stages of illness with or without antipsychotic treatment, and these alterations are evident in the central nervous system as well as in peripheral tissues and fluids of patients with schizophrenia.

**Table 1:** Summary of evidence for altered glycosylation in schizophrenia and/or in association with antipsychotic administration.

GWAS of patients over the progression of illness^22^	Early stage: carbohydrate metabolism, lipid metabolism, protein localization, protein transport/modification	
	Intermediate stage: biopolymer glycosylation, protein amino acid glycosylation, glycoprotein biosynthesis, glycosylation/glycoprotein, lipid metabolism, protein localization, protein transport/modification	
	Late stage: glycosylation/glycoprotein, lipid metabolism, protein localization, protein transport/modification	
GWAS of patients on antipsychotic medication^23^	Schizophrenia patients on typical antipsychotics versus controls: ER, ER-Golgi transport, Golgi transport	
	Schizophrenia patients on atypical antipsychotics versus controls: stress response, protein binding	
	Schizophrenia patients on typical versus atypical antipsychotics: cellular lipid metabolism, lipid biosynthesis, lipid metabolism	
Canonical pathway analyses^70^	Schizophrenia patients on typical antipsychotics versus controls: N- and O-linked glycan biosynthesis, glycosphingolipid biosynthetic pathways	
*Glycoprotein expression levels in schizophrenia*		
Urine (males)	Lower 24-h secretion of urinary glycoproteins^24^	
Serum	Increased concentration of protein-bound carbohydrates^26^	
	Increased levels of α2- and β-globulin glycoproteins^26^	
*Monosaccharide composition of glycoprotein glycans in schizophrenia*		
Urine (males)	Presence of rhamnose detected (trace amounts detected in only a few non-psychiatrically ill subjects)^24^	
	Increased ratio of glucosamine:galactosamine^24^	
	Decreased ratios of Fuc: Neu5Ac, Fuc: hexose, hexose: hexosamine, and Fuc: hexosamine^24^	
Urine	(acidic glycopeptide and oligosaccharide fraction)	Decreased hexose levels^25^
		Increased ratio of Gal: Man^25^
	(basic, neutral, or slightly acidic glycopeptide and oligosaccharide fraction)	Decreased rhamnose^25^
		Increased Fuc^25^
Serum	Increased Glc and arabanose^26^	
	Altered monosaccharide composition of α2- and β-globulin glycans^26^	
Serum (age 13–17)	Increased Fuc, Man, Glc, Gal, Neu5Ac, glucosamine, and galactosamine^27^	
	Increased total hexose and hexosamine levels^27^	
*N*-*glycosylation of neurotransmission associated proteins in schizophrenia*		
ACC	Smaller *N*-glycans on EAAT1 monomer^28^	
DLPFC	Smaller *N*-glycans on EAAT2 multimer^28^	
	Decreased ratio of EndoH sensitive versus insensitive GluA2^29^	
	Decreased binding of ConA to GluA2^29^	
	Larger immature *N*-glycans on GluK2^30^	
STG	Smaller immature *N*-glycans on GABRA1^31^	
	More immature *N*-glycosylation of GABRB1 49 kDa isoform^31^	
	Altered *N*-glycosylation of GABRB2 concurrent with increased molecular mass of GABRB2 isoforms^31^	
Gene and protein expression of glycosylation enzymes in schizophrenia (see Table 2)		
*Altered glycosylation enzyme activity in schizophrenia*		
Plasma	Increased α-2,6-sialyltransferase activity^109^	
*Glycomic differences in first onset schizophrenia patients*		
Serum	(high abundance protein fraction)	Peak (H6) containing 5 *N*-glycan structures is increased in male patients and decreased in female patients relative to non-psychiatrically ill subjects^80^
	(low abundance protein fraction)	Peaks (U23 and U19) are increased in male schizophrenia patients^80^
	Altered *N*-glycans contain sialylated *N*-acetyllactosamine motifs^80^	
CSF	Peaks (C17, C18, C20) are decreased in schizophrenia^80^	
	Peak (C3) is increased in female patients and decreased in male patients relative to non-psychiatrically ill subjects^80^	
*Gene expression of glycosylation enzymes related to antipsychotic administration*		
Liver	Increased expression of B4GALT1 in patients treated with atypical antipsychotics^23^	
*Glycomic and glycoprotein differences related to antipsychotic administration (olanzapine) in schizophrenia*		
Serum	Peak (16) containing a disialylated digalactosylated biantennary *N*-glycan is increased^81^	
	Peak (20) containing 3 disialylated *N*-glycans is decreased^81^	
	α1 acid glycoprotein (AGP) has increased peak 16 and decreased peak 24 (containing multiple tetra-antenarry *N*-glycans)^81^	
	Decreased levels of non- and mono-galactosylated glycans concurrent with increased levels of digalactosylated glycans^81^	
	Decreased level of mono-sialylated glycans concurrent with an increased level of disialylated glycans^81^	
*Altered NCAM polysialylation in schizophrenia*		
Hippocampus	Fewer PSA-NCAM-immunoreactive cells^88^	
	Decreased polysialylation of NCAM^88^	
DLPFC	Decreased PSA-NCAM in cortical layers IV and V^89^	
CSF	105–115 kDa NCAM isoform represents a cleavage product of non-polysialylated NCAM (cNCAM), and cNCAM is increased^95^	
	Cleaved NCAM level is positively correlated with ventricular volume changes in brain and patient score on the Scale for Assessment of Positive Symptoms (SANS)^95^	
*ST8SIA2 association with schizophrenia*		
Genomic DNA	SNPs/SNP haplotypes of ST8SIA2 are associated with schizophrenia risk^97,99–101,103^

**Table 2:** Glycosylation enzyme gene and protein expression alterations in schizophrenia brain.

Brain region	BA46			BA9/46			BA22	
Reference	^70^			^53^	^52^	^55^	^72^	^54^
Glycosylation enzymes	Gene array	Gene RT-PCR short-term DOI	Gene RT-PCR long-term DOI	Gene array	Protein	Protein	Protein	Protein
AGA	↓			↔				
B3GLCT (B3GALTL)				↑			↔	
B3GNT2	↔			↔	↔			
B3GNT3	↔			↔	↔			
B3GNT4	↔			↑				
B3GNT8 (B3GNTL1)				↑	↓			
B4GALT1	↔			↔			↓	
B4GALT2				↑				
B4GALT3				↑				
B4GALT6	*	↓	↔					
C1GALT1	↔			↑			↔	
C1GALT1C1	↔			↑			↔	
EDEM1				↔		↔		
EDEM2				↑		↑		
EDEM3				↔		↔		
FUCA2				↑				↔
FUT11	↔			↑				↔
FUT6	*							
FUT8	↔			↑				↓
GAL3ST1	↔	↓	↔					
GALC	↔	↓	↔					
GALNT10				↑				
GALNT2	↔			↑			↔	
GALNT3				↑				
GALNT4				↑				
GALNT5	*							
GALNT7	↔			↔			↔	
GALNT16 (GALNTL1)	↔			↔			↓	
GALNTL5	↔			↑			↔	
GANAB				↔		↔		
GCNT3				↑				
MAN1A1				↑				
MAN1B1				↑		↔		
MAN2A2	*			↔				
MGAT1	↔			↑	↔			
MGAT2				↔				
MGAT3	*			↑				
MGAT4A	↔			↑	↓			
MGAT4B				↔	↔			
MGAT4C				↔	↔			
MGAT5	↔			↑	↔			
MGAT5B				↑	↔			
MOGS				↑		↔		
NEU1				↑				
NEU2				↑				
NEU3				↑				
OGT				↑				
PDIA3 (ERp57)						↔		
POFUT1	↔			↑				↔
POFUT2	↔			↔				↑
ST3GAL1				↑				
ST3GAL2	*			↑				
ST6GAL1	*			↑			↔	
ST8SIA3				↑				
ST8SIA6				↑				
UGGT1				↔		↔		
UGGT2				↑		↑		
UGT8	↓	↓	↔					

Glycosylation enzymes with reported gene and protein expression differences in schizophrenia brain are summarized from refs. ^52–55,70,72^ The direction of gene expression differences in schizophrenia for some enzymes were not specified and are indicated with an asterisk (*). Genes which do not demonstrate differential expression in schizophrenia are listed only if both gene and protein expression of the enzyme have been measured. Symbols: increased expression (↑), decreased expression (↓), no expression difference detected (↔).

**Table 3:** Summary of evidence for altered lipidation in schizophrenia and/or in association with antipsychotic administration.

*Altered expression of myristoylated substrates in schizophrenia*	
DLPFC	Decreased protein expression of MARCKS and pMARCKS^112^
*Expression of prenylation-associated enzymes in schizophrenia*	
DLPFC	Decreased protein expression of FNTA, PGGT1B, and RABGGTB^118^
*Protein S-palmitoylation in schizophrenia*	
DLPFC	Decreased total levels of palmitoylated protein and reduced levels of palmitoylated protein within specific molecular mass ranges^126^
	Decreased palmitoylation of VGLUT1, Ras, and MBP^126^
*S-palmitoylation-associated enzyme activity in schizophrenia*	
Serum	Increased enzyme activity of PPT1^130^
	PPT1 activity is positively correlated with Positive and Negative Syndrome Scale (PANSS) scores for positive, negative, general, and S scales^130^
*DHHC enzyme (protein acyltransferase) gene expression in schizophrenia*	
GWAS	ZDHHC18 and ZDHHC5 were among the top 5 genes significantly associated with schizophrenia^131^
	Loss-of-function mutation of ZDHHC5 identified within schizophrenia-associated loci^132^
*22q11 deletion syndrome is associated with schizophrenia risk*	
Reviews	22q11 deletion syndrome implicated as a genetic subtype of schizophrenia^133,135,136^
Targeted gene analysis	~2% of adult caucasians with schizophrenia exhibit 22q11 deletion^134^
SNP analysis	ZDHHC8 (KIAA1292) gene is located in the 22q11 susceptibility locus and implicated in schizophrenia^137^
*SNP rs175174 in the ZDHHC8 gene is associated with schizophrenia risk*	
SNP analysis	Increased transmission of the A allele of rs175174 in females with schizophrenia^141,143^
Meta-analysis	Weakly supports rs175174 as a schizophrenia risk factor in East Asian populations^138^
SNP analysis	Interaction of the rs175174 and rs5992403 (UFD1L) is associated with schizophrenia age of onset^139^
Genotype/MRI	GG-genotype at rs175174 is associated with reduced gray matter volume in the frontal lobe, but is not specific to schizophrenia^140^
*Hh family protein lipidation in schizophrenia*	
GWAS	SNP rs7527939 in the HHAT gene was the strongest indicator of schizophrenia risk in Bulgarian subjects^149^
*Ghrelin octanoylation associated with antipsychotic administration*	
Plasma	Octanoylated (active) ghrelin levels are unchanged after 4, 8, 12, and 16-weeks olanzapine treatment, despite reductions of total ghrelin at 8, 12, and 16 weeks^157^

## Early evidence of glycosylation abnormalities in schizophrenia

One of the first glycoprotein assessments in schizophrenia reported reduced glycoprotein expression in urine samples from male schizophrenia patients, as well as abnormal monosaccharide composition of glycoproteins.^24^ Using available technologies at the time, it was determined that the ratios of monosaccharide components of glycoproteins (glucosamine:galactosamine, Fuc:Neu5Ac, Fuc:hexose, hexose:hexosamine, and Fuc:hexosamine) were altered in schizophrenia and are consistent with abnormal glycan composition.^24^ Another study evaluated patient urine following fractionation into samples of (I) basic, neutral, or slightly acidic glycoproteins and oligosaccharides, (II) acidic glycopeptides, and (III) glycosaminoglycans. This study, in both male and female subjects, failed to replicate the prior findings, but did find that hexose levels and the ratio of Gal:Man were reduced in fraction II and increased Fuc was identified in fraction I in schizophrenia.^25^ Although the two studies reported dissimilar results, both indicate that processing of secreted glycoproteins is abnormal in schizophrenia.

In patient blood serum, analysis of monosaccharide levels from a glycoprotein enriched sample found that the concentration of glycoprotein carbohydrates was higher in schizophrenia. The monosaccharide composition of protein-bound carbohydrates was also different between patients and comparison subjects.^26^ This study also used a substrate-specific approach and examined the abundance of α2- and β-globulin, reporting a relative increase in expression of these glycoproteins as well as altered monosaccharide composition of their attached glycans.^26^ Serum glycoprotein assessments have also been performed in children with schizophrenia (age 13–17), and an increase in glycoprotein levels was identified.^27^ Based on these early studies, abnormal glycoconjugate metabolism in schizophrenia was suggested to be influenced by an immunological response arising from the psychological distress of the disorder.^27^

## Evidence of altered *N*-glycosylation of neurotransmission-associated proteins in postmortem schizophrenia brain

Abnormal *N-*glycosylation of neurotransmission-associated proteins has been reported in schizophrenia, especially for proteins associated with glutamate and γ-aminobutyric acid (GABA) signaling. Differences of *N-*glycosylation of excitatory amino acid transporters (EAAT) 1 and 2, α-amino-3-hydroxy-5-methyl-4-isoxazolepropionic acid (AMPA) receptor subunit 2 (called GluR2, GRIA2, or GluA2), kainate receptor subunit 2 (called GluR6, GRIK2, or GluK2), and the GABA type A receptor (GABA_A_R) α1, β1, and β2 subunits have all been reported in schizophrenia.^28–31^ Although *N-*glycosylation differences of receptors and/or transporters for other neurotransmitter systems have not yet been reported, this is likely due to a lack of glycobiological assessments of these molecules rather than a failure to identify differences in other neurotransmitter systems.

**Fig. 2:**
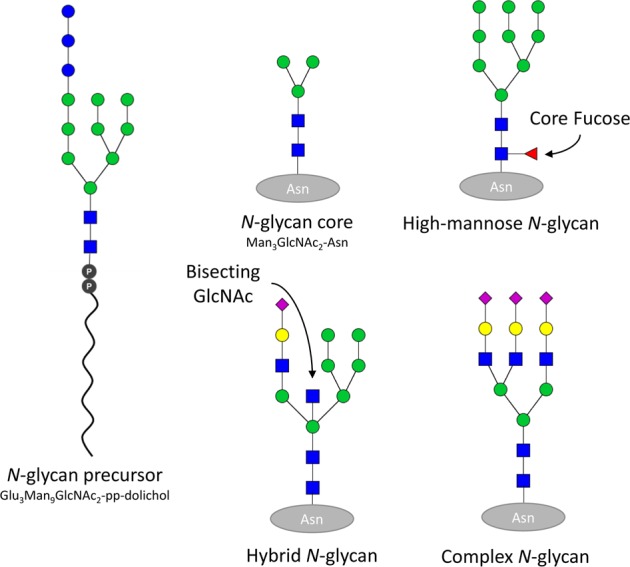
*N-*glycan structures. Schematics of representative *N-*glycan structures expressed in mammals. The *N-*glycan precursor oligosaccharide (Glu_3_Man_9_GlcNAc_2_-pp-dolichol) is attached co- or post-translationally to proteins containing the consensus sequence Asn-Xaa-(Ser/Thr), where Xaa is any amino acid except proline. Substrate proteins are represented as gray ovals and example *N-*glycan structures are represented following established symbol nomenclature conventions.^163^ Subsequent modification of *N-*glycans by the transfer or cleavage of monosaccharide units following attachment to the protein can produce high-mannose, hybrid, or complex *N-*glycans. The simplest possible *N-*glycan consists of Man_3_GlcNAc_2_-Asn, and this represents the *N-*glycan core structure that is present in all *N-*linked glycans. Some common substructures, such as core fucose or bisecting GlcNAc provide important spatial constraints within the glycan structure and can influence subsequent glycan processing steps. Structures depicted here are representative examples of *N-*glycan subtypes. Hundreds of possible *N-*glycan structures can be synthesized by mammals and the specific glycan structures synthesized by any particular cell is determined by the spatiotemporal colocalization of enzyme, nucleotide-sugar donor, and substrate protein.

The most common form of *N*-glycosylation is initiated in the ER when the oligosaccaryltransferase complex transfers the carbohydrate portion of a precursor molecule (Glc_3_Man_9_GlcNAc_2_-pp-dolichol; Fig. 2) onto an asparagine (Asn) residue of a growing polypeptide chain within the consensus sequence Asn-Xaa-Ser/Thr (threonine), where Xaa is any amino acid except proline.^32^ Although commonly referred to as a PTM, most *N*-glycosylation occurs co-translationally and provides a spatial constraint to facilitate formation of intrapeptide bonds.^33,34^ Proteins can have one or many *N*-glycosylation consensus sites, and the pattern of *N-*glycan structures and consensus site occupancy may be developmentally regulated by the cell/tissue type and species in which the substrate is expressed.^11,12^ For multimeric protein complexes such as neurotransmitter receptors, the presence or absence of *N-*glycans at specific consensus sites can also affect the stoichiometry and assembly of complexes, as well as the functional properties of assembled multimers.^34–36^

Endoglycosidase H (EndoH), which cleaves high-mannose immature *N-*glycans, or protein *N-*glycosidase F (PNGaseF), which cleaves mature *N*-glycans, are glycosidases typically used in deglycosylation shift assays to assess protein *N-*glycosylation status. These assays can identify differences in the relative size of *N-*glycans attached to a target protein by comparing the kilodalton (kDa) shift of enzyme-treated versus control-treated samples, or can give an estimation of the relative amount of protein that is *N-*glycosylated by comparing the expression of enzyme-sensitive versus enzyme-insensitive glycoforms in enzyme-treated samples.

EAAT1 and EAAT2, which modulate glutamate uptake from the synaptic cleft and extrasynaptic extracellular matrix into astroglia, are abnormally *N-*glycosylated in schizophrenia anterior cingulate cortex (ACC) and dorsolateral prefrontal cortex (DLPFC), respectively.^28^ In the ACC, the EAAT1 monomer demonstrates a smaller shift after PNGaseF treatment, indicating that EAAT1 *N-*glycans are of smaller molecular mass in schizophrenia, whereas EAAT2 demonstrates a reduced PNGaseF shift of its multimeric form in the DLPFC in schizophrenia. This suggests either a less complex *N-*glycan structure or altered *N-*glycan composition on the EAAT1 monomer and EAAT2 multimer in schizophrenia. Reduced perisynaptic glutamate buffering, leading to glutamate spillover and loss of signaling specificity are molecular features implicated in schizophrenia pathophysiology.^2,37^ As the *N-*glycosylation status of EAAT1 and EAAT2 regulates their expression in the plasma membrane,^38^ smaller *N-*glycans on these molecules may contribute to glutamatergic signaling alterations in this illness by impacting the ability of astroglia to modulate extracellular glutamate levels.^28,37,39^

Lectin affinity isolation methods similar to antibody-based immunoprecipitation can also be used to assess glycosylation differences on substrate proteins. Instead of using an antibody which recognizes a particular protein epitope to capture target proteins, a lectin or panel of lectins (glycan-binding proteins) that recognize specific glycan conformations can be used to capture proteins adorned by target glycan structures. These methods provide an indication of the monosaccharide composition of glycans based on the pattern of lectins that bind the target protein.

Most AMPA receptors are tetrameric receptors assembled as a “dimer of dimers”, and the most abundant in the nervous system are GluA2-containing receptors.^40,41^ In schizophrenia, the GluA2 AMPA receptor subunit shows a reduced ratio of EndoH sensitive:EndoH insensitive glycoform expression as well as reduced binding to the lectin Concanavalin A (ConA).^29^ AMPA subunit glycosylation patterns are highly conserved in evolution,^42^ and AMPA receptor subunit *N-*glycosylation status is a key mediator of receptor assembly, stoichiometry, cell surface expression, receptor-signaling properties, and receptor trafficking and modulation by associated proteins.^43–49^ Given that EndoH cleaves only high-mannose *N-*glycans and ConA is known to specifically bind high-mannose *N-*glycans, these findings suggest abnormal immature *N-*glycan processing of the GluA2 subunit in the ER. Reduced high-mannose glycans on GluA2 in the absence of altered GluA2 protein levels could suggest accelerated exit of GluA2-containing receptors from the ER. GluA1 subunits, which are most often assembled into GluA1/2 tetrameric receptors, demonstrate altered subcellular distribution along the forward trafficking pathway, supporting the interpretation that some AMPA receptor subpopulations may be exported from the ER more rapidly in schizophrenia.^50^ Although AMPA subunit expression in the ER compartment is reported to be intact in schizophrenia,^51^ the glycosylation state of AMPA subunits was not specifically assessed.

Examination of glycosylation enzyme expression in schizophrenia has found altered protein levels for enzymes that act on a variety of monosaccharides. Recent reports provide evidence for abnormal protein expression of a variety of functional categories of glycosylation enzyme in schizophrenia brain.^52–55^ Interestingly, reduced expression of the GlcNAc transferases B3GNT8 and MGAT4A has been identified.^52^ Defects in MGAT4A expression can influence the degree of glycan branching on substrate proteins and consequently their residence time in the plasma membrane. This defect was suggested as a potential mechanism for increased localization of AMPA receptor subunit GluA1 to early endosomes in schizophrenia.^50,52^

Taken together, these reports suggest that GluA2 subunits which are not *N-*glycosylated are retained in the ER, while *N-*glycosylated GluA2 is preferentially assembled into intact GluA1/2 receptors which are then rapidly exported from the ER and trafficked to the cell surface. AMPA receptor subunit glycosylation state and effects of AMPA subunit glycosylation on cellular function in the central nervous system is not simply a matter of whether or not a particular subunit is glycosylated. Patterns of *N-*glycosylation consensus sequence occupancy and the expression of specific glycan structures are also known to influence AMPA receptor assembly, surface expression, and signaling properties^43–45,47,56^ suggesting that abnormal glycosylation or glycan composition is a likely component of AMPA-associated neurotransmission alterations in the illness.

In addition to the potential role of altered *N-*glycosylation of the EAAT1 and EAAT2 glutamate transporters and the GluA2 subunit in glutamatergic signaling abnormalities in schizophrenia, abnormal *N-*glycosylation of kainate receptor subunit GluK2 was also identified.^30^ GluK2 showed a larger molecular mass shift after treatment with EndoH, which indicates a larger immature *N-*glycan on GluK2 in the disorder. Larger high-mannose glycans suggest that the immature *N-*glycan has not undergone extensive processing and GluK2 progression along the forward trafficking pathway may be impaired. GluK2 is a major mediator of kainate receptor cell surface expression on both pre- and post-synaptic membranes, and kainate receptors are important regulators of neuronal metabotropic signaling.^57^ Both AMPA and kainate receptors can be modulated by endogenous lectin-like molecules called galectins, which bind to specific galactosylated glycan structures and alter the desensitization properties of the receptor.^49^ It is possible that in schizophrenia, abnormalities of AMPA and kainate receptor *N-*glycosylation impair galectin–receptor interactions and contribute to abnormal neurotransmission.^48,49^

Abnormal protein *N-*glycosylation in schizophrenia is not exclusive to the glutamate system. Altered *N-*glycosylation of GABA_A_R α1, β1, and β2 subunits has been reported in the superior temporal gyrus (STG) in schizophrenia. GABA_A_R α1 and β1 subunits exhibit abnormal immature *N-*glycosylation, while the β2 GABA_A_R subunit had alterations of total *N-*glycosylation.^31^ After EndoH treatment, the α1 subunit demonstrated a reduced molecular mass shift in schizophrenia, indicating a smaller immature *N-*glycan. The β1 subunit, on the other hand, reflects an abnormal ratio of subunit isoforms following EndoH deglycosylation. This suggests that some β1 isoforms may be preferentially *N-*glycosylated in schizophrenia. Unlike α1 and β1, The GABA_A_R β2 subunit did not exhibit abnormalities in schizophrenia following EndoH treatment, but instead a larger molecular mass shift was identified after treatment with PNGaseF, which suggests that *N-*glycans on the β2 subunit may be larger or more complex in schizophrenia.^31^

Most GABA_A_Rs are composed of two α, two β, and one γ subunit.^58^ An investigation of GABA_A_R subunit subcellular localization in schizophrenia found altered expression of specific β1 and β2 subunit isoforms and glycoforms in biochemical fractions enriched for either ER membranes or synapses. Differences identified at either end of the forward trafficking pathway (ER compartment versus synapses) suggest that an *N-*glycosylated GABA_A_R β2 subunit with a larger or more complex *N-*glycan may be preferentially incorporated into synaptically targeted GABA_A_Rs in schizophrenia, and dysglycosylation of GABA_A_R subunits may contribute to abnormalities of inhibitory signaling in this illness.^31,59^

## Evidence of glycan processing pathway dysregulation in schizophrenia

**Fig. 3:**
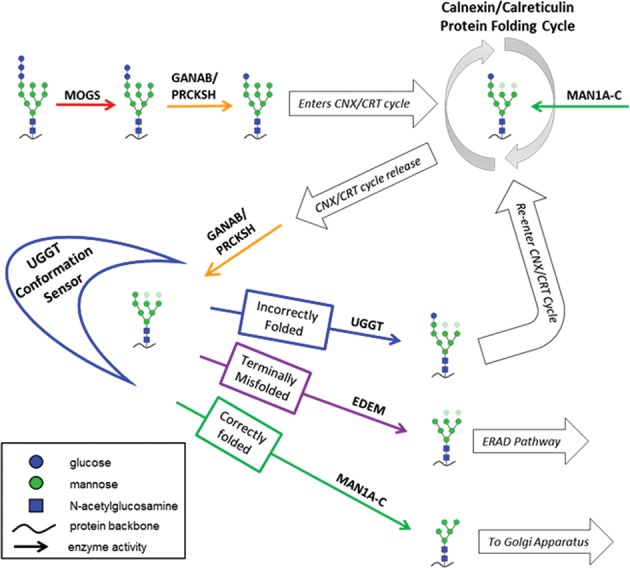
Step-wise *N-*glycoprotein processing in the endoplasmic reticulum (ER). Schematic representation of immature *N-*glycoprotein processing in the ER. After the *N-*glycan oligosaccharide precursor is attached to a protein substrate, α-glucosidase I (MOGS, red) cleaves the most terminal glucose. Cleavage of the next glucose by α-glucosidase II (GANAB, orange) is regulated by glucosidase II subunit β (PRCKSH, orange), and this intermediate *N-*glycan conformation allows the *N-*glycoprotein to interact with chaperone proteins in the calnexin–calreticulin (CNX/CRT) protein folding cycle. While interacting with molecules in the CNX/CRT cycle, ER-localized α-mannosidase I (MAN1A-C, green) cleaves mannose residues. After *N-*glycoprotein release from the CNX/CRT protein-folding cycle, GANAB/PRCKSH cleaves the final terminal glucose to allow interaction with UDP-glucose:glycoprotein glucosyltransferases (UGGT1/2, blue). UGGT proteins have two main functions: (1) to act as conformation sensors that identify incompletely folded or misfolded proteins and, if the protein is improperly folded, (2) to catalyze the re-addition of a terminal glucose to permit return to the CNX/CRT protein folding cycle. Correctly folded *N-*glycoproteins are released from the UGGT conformation sensor and can be further modified by MAN1A-C prior to forward trafficking to the Golgi apparatus for maturation into a complex *N-*glycan structure. If a protein fails to attain the correct tertiary structure and is terminally misfolded, ER degradation-enhancing mannosidases (EDEM1-3, purple) interact with the glycoprotein after release from the UGGT conformation sensor and remove mannose residues to produce a Man_6-8_GlcNAc_2_-polypeptide structure which is exported from the ER to be degraded via the ER-associated degradation pathway.

Following *N-*glycan attachment, carbohydrate structures are modified in the ER in a specific step-wise manner (Fig. 3), and the specific carbohydrate composition of the glycan structure reflects whether the nascent peptide chain has the necessary intramolecular interactions to achieve a correct tertiary protein structure.^12,60^ Some enzymes with abnormal protein expression levels in schizophrenia are mediators of glycoprotein folding and ER quality control. When immature *N*-glycoproteins are critically misfolded, they are targeted to the ER-associated degradation (ERAD) pathway by the activity of glycosylation enzymes that have dual functions as recognition molecules.^34^ UGGT (UDP-glucose:glycoprotein glucosyltransferase) proteins serve as recognition molecules that identify when an *N-*glycoprotein has achieved the appropriate tertiary conformation.^12,34,61^ After interacting with UGGT proteins in the lumen of the ER, *N-*glycoproteins are either released for export to the Golgi apparatus or, if improperly folded, UGGTs re-attach a terminal Glc residue which targets the glycoprotein back into the calnexin–calreticulin (CNX/CRT) protein folding cycle (Fig. 3).^34,61,62^ ER degradation enhancing α-mannosidase (EDEM) proteins, on the other hand, cleave Man residues from *N*-glycans in the ER, and specific Man substructures provide an indication of how long the substrate polypeptide chain has spent in the CNX/CRT cycle.^12,34,63–65^ When a particular high-mannose structure (Man_6-8_GlcNAc_2_-Asn) is achieved by the sequential removal of terminal Man by the mannosidase activity of EDEM proteins, the substrate is identified as terminally misfolded and targeted to the ERAD pathway (Fig. 3).^34,64^ Both UGGT2 and EDEM2 exhibit increased gene and protein expression in schizophrenia DLPFC,^53,55^ which may reflect enhanced targeting of *N*-glycoproteins to ERAD or represent a mechanism that contributes to accelerated exit of some proteins from the ER in schizophrenia.

After correctly folded *N-*glycoproteins are exported to the Golgi apparatus, *N-*glycans may be further modified by enzymes that attach or cleave specific monosaccharides, glycan structures, and/or small molecules (e.g. phosphate, sulfate).^12,66,67^ *N-*glycans can be modified into highly complex branched structures with sub-structural elements that confer specific properties to the *N-*glycoprotein (Fig. 2). Many transmembrane proteins are *N-*glycosylated and when inserted into the cell membrane the *N-*glycan faces the extracellular space.^32^ The presence or absence of an extracellular-facing *N-*glycan (or a sub-structural feature or glycoepitope within an *N-*glycan) can impact the ligand-binding affinity of receptors and/or the kinetics of channel opening in a substrate-specific manner. Extracellular *N-*glycans can also influence cell–cell interactions, features of the extracellular matrix, and provide immune recognition signals.^10–12,68,69^

Building on a prior GWAS study,^22^ a GLYCOv2 Affymetrix GeneChip developed by the Consortium for Functional Glycomics was used to more specifically assess dysregulated interaction networks associated with glycosylation pathways in the DLPFC in schizophrenia. Canonical pathways analyses found *N*- and *O-*linked glycan biosynthesis as well as ganglioseries, lactoseries, and globoseries glycosphingolipid biosynthetic pathways enriched in schizophrenia.^70^ A more recent exploratory study of mRNA expression of glycosylation-associated enzymes in DLPFC using the Qiagen Human Glycosylation RT^2^ Profiler PCR Array assessed transcript expression of enzymes from multiple glycosylation pathways (including glycosphingolipid metabolism) and found differential expression of 36 enzymes in schizophrenia, 35 of which participate in either or both *N*- and *O-*linked protein glycosylation.^53^ Protein levels of several glycosylation associated enzymes have been assessed in postmortem brain and, although protein alterations in schizophrenia do not necessarily reflect reported transcript level differences (Table 2), the molecular functions and pathways implicated by gene and/or protein alterations are similar.

## Evidence of altered *O-*linked glycosylation in schizophrenia

*O-*glycosylation differs from *N-*glycosylation in that there exist a variety of *O-*glycan subtypes with variable features and functions.^10–12^ *O-*glycans are designated by the monosaccharide attached to the protein backbone. With a few exceptions, *O-*glycosylation generally does not require a specific consensus sequence for glycan attachment. In contrast to *N-*glycan processing, the potential extension of an *O-*linked monosaccharide into a multimeric glycan structure can differ significantly between substrates, and can vary between identical structural domains of the same protein.

The most abundant *O-*glycan subtype is *O-*GalNAc, also called mucin-like *O-*glycosylation, which is synthesized during protein processing in the Golgi apparatus^71^ These glycoproteins typically serve a protective function by acting as a carbohydrate cushion at the cell surface and participating in immune signaling of all cell types.^69,71^ The enzyme GALNT16 is involved in the substrate-specific synthesis of O-GalNAc glycans and protein expression of this enzyme has been found reduced in schizophrenia STG.^72^

**Fig. 4:**
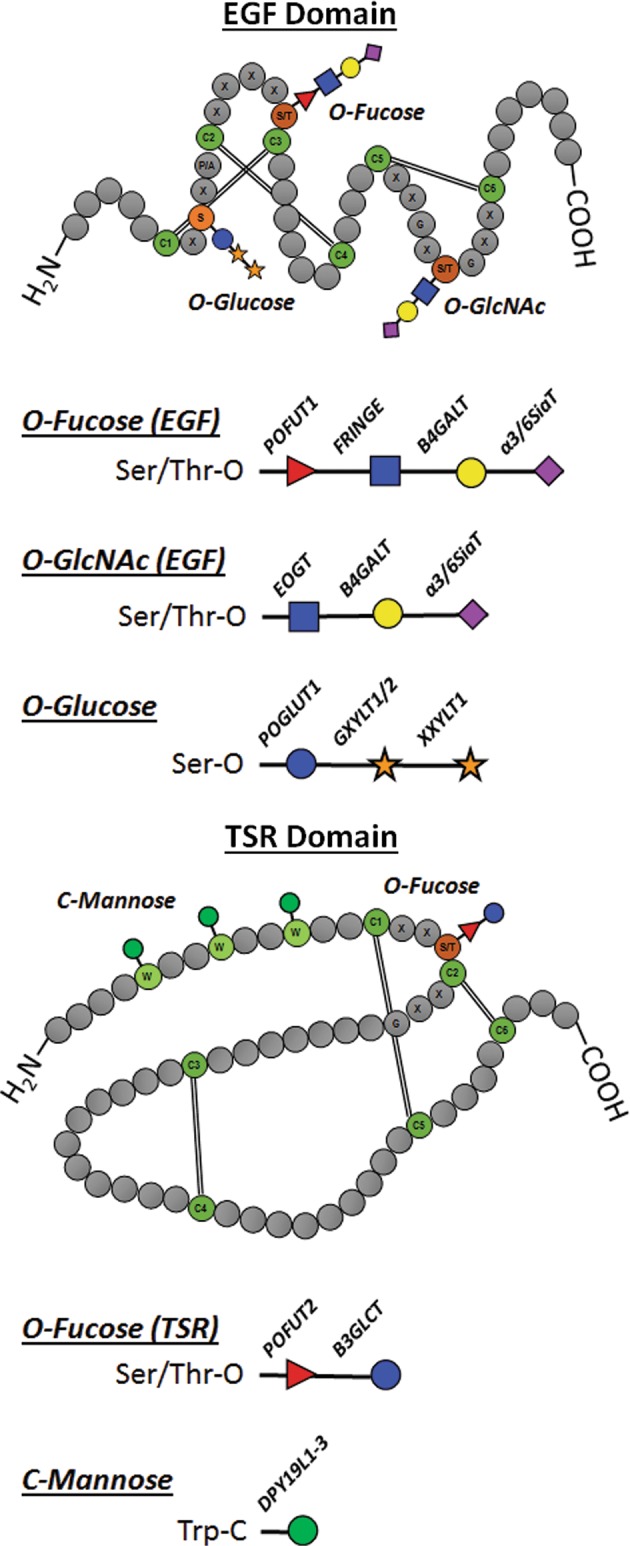
Cysteine knot *O-*glycosylation. Schematics of the amino acid sequences and glycan structures associated with cysteine knot formation of EGF and TSR domain-containing proteins. Cysteines involved in di-sulfide bond formation and glycosylation sites are depicted by colored circles, other amino acids are depicted as gray circles. Disulfide bonds are indicated by double lines. Consensus sequences are indicated with the appropriate amino acid abbreviation listed in the gray circle; X indicates unspecified amino acid residues that form part of an established consensus sequence. Glycans are represented as the most elaborate structure which can be formed or predicted at each glycosylation site. Glycan structural diagrams list the specific enzyme or glycosyltransferase family which can catalyze the addition of each carbohydrate.

Some *O-*glycan subtypes precisely modify proteins containing specific structural elements, such as the cysteine knots in epidermal growth factor (EGF)-like or thrombospondin-like repeat (TSR) domains. *O-*fucosylation and/or *O-*glucosylation of EGF and EGF-like domain-containing proteins occurs in the ER, and stabilizes the amino acid chain to facilitate correct disulfide bond formation to produce the knot structure (Fig. 4).^33,73^ Similar to EGF-lke domains, TSR domain modification by *O-*fucose in the ER by the enzyme POFUT2, which was found increased in schizophrenia STG,^54^ stabilizes the polypeptide for cysteine-knot formation.^74,75^ When POFUT2-mediated *O-*fucosylation of TSR domains is impaired substrate proteins are directed to a non-canonical ERAD pathway,^73,74^ thus increased expression of POFUT2 may indicate another potential mechanism of enhanced ER quality control in schizophrenia.

Interestingly, many TSR domain-containing proteins are important regulators of cell–cell interactions or modify the composition of the extracellular matrix.^76^ *O-*fucosylation and the proper formation of the cysteine knot on TSR domain-containing proteins has been shown to be necessary for their secretion into the extracellular matrix.^73,74,77,78^ ADAMTS (a disintegrin and metalloproteinase with thrombospondin motifs) proteins are key substrates of POFUT2 that are associated with synaptic plasticity, and are important regulators of extracellular matrix remodeling and perineuronal net formation, features of the tetrapartite synapse which have been reported to be abnormal in schizophrenia brain.^79^

## Evidence of altered glycan structures in schizophrenia

In addition to finding abnormal expression of specific glycosylation associated enzymes, glycomic investigations of blood serum and CSF samples indicate that specific glycan structures are abnormal in antipsychotic-naïve schizophrenia patients relative to non-psychiatrically ill subjects.^80^ Patient serum was separated into low-abundance and high-abundance serum protein fractions, and released *N-*glycans from each fraction were analyzed by normal phase high performance liquid chromatography (NP-HPLC). NP-HPLC peaks that were differentially expressed in schizophrenia serum contained seven complex *N-*glycan structures with sialylated *N-*acetyllactosamine motifs. In CSF, differentially expressed NP-HPLC peaks contained 11 *N-*glycan structures, and the pattern of expressed *N-*glycans can distinguish between patients and controls with high predictive power.^80^ Another report identified decreased gene expression of MAN2A2, MGAT3, ST6GAL1, and ST3GAL2 in schizophrenia brain, and these enzymes are necessary for the synthesis of some of the glycan structures found abnormally expressed in schizophrenia CSF.^70,80^ The concordance between finding abnormal enzyme expression in postmortem schizophrenia brain in conjunction with evidence that enzyme product structures are altered in peripheral fluids of living patients suggests that these are not transient abnormalities associated with dietary or lifestyle habits, but instead represent persistent defects of cellular glycan processing.

Glycomic differences between schizophrenia patients and comparison subjects, as well as changes in the serum glycome of antipsychotic-naïve patients after 6 weeks of olanzapine treatment, have been reported.^80,81^ Olanzapine administration results in a relative increase in the degree of galactosylation and sialylation of serum *N-*glycans without a concurrent change in glycan branching.^81^ This finding is consistent with increased gene expression of the galactosyltransferase B4GALT1 identified in liver following administration of second generation antipsychotics.^23^ Increased expression of the micro-RNA miR-124-3p has been shown to inhibit B4GALT1 expression^82^ and upregulation of miR-124-3p in drug-free schizophrenia patients is reduced following 12 weeks of second generation antipsychotic administration.^83^ A recent study in schizophrenia postmortem STG found B4GALT1 protein levels reduced, but the patients included in the study were elderly subjects who had primarily been treated with first generation antipsychotics.^72^ Together, these reports suggest that both first and second generation antipsychotic medications alter glycosylation pathway activity but may do so via different mechanisms.

A specific glycan structure that is highly regulated during neurodevelopment is polysialic acid (PSA). Polysialylation is the addition of up to 400 Neu5Ac residues in an α-2,8-linkage onto a terminal α-2,3/6-Neu5Ac. The mechanisms and role of (poly)sialylation in the nervous system have been extensively reviewed.^84^ The PSA modification was initially believed to occur exclusively on *N-*glycans of neural cell adhesion molecule 1 (NCAM1). PSA modification of the polysialyltransferases ST8SIA2 and ST8SIA4, SynCAM1, neuropilin-2, voltage-sensitive sodium channel α subunit, and CD36 scavenger receptor in human milk have also been identified, but these substrates have not been as extensively studied as polysialylated NCAM1 (PSA-NCAM).^84,85^ PSA-NCAM is an important mediator of human neurodevelopment and synaptic plasticity, particularly during embryonic development. In later stages of neurodevelopment, PSA-NCAM is less abundant but continues to exhibit diffuse expression throughout the brain with higher levels detected in regions of high synaptic plasticity, such as the hippocampus.^86^ PSA-NCAM has also been shown to be a mediator of activity-driven morphological changes of both neurons and astroglial processes that occur during synaptic remodeling.^87^

Evidence for reduced expression of PSA-NCAM without a concurrent change in total NCAM1 expression in schizophrenia was first reported in the hippocampus.^88^ Reduced expression of PSA-NCAM was also identified in DLPFC layers IV and V and in the medial prefrontal cortex (mPFC) of patients. In DLPFC layer IV and mPFC, reduced PSA-NCAM was concurrent with reduced GAD67 expression, suggesting that these alterations may contribute to abnormal inhibitory neurotransmission in schizophrenia.^89^ Conversely, PSA-NCAM levels in the neuropil of the amygdala and the STG of schizophrenia subjects were not different from non-psychiatrically ill subjects.^90,91^ Together, these reports indicate that PSA-NCAM associated impairments are brain-region and cortical layer specific in later stages, but do not exclude the possibility that NCAM1 polysialylation abnormalities earlier in development may contribute to the pathophysiology of this disorder.

Several studies have evaluated NCAM1 expression in patient CSF and found increased expression of a secreted form of the molecule.^92–94^ Characterization of the CSF-associated NCAM1 isoform revealed that this molecule results from the extracellular cleavage of the 180 kDa isoform of NCAM1 and that the addition of PSA chains to the 180 kDa NCAM1 isoform is protective against proteolysis; thus, cleaved NCAM1 (cNCAM) in the CSF represents an indirect measure of non-polysialylated 180 kDa NCAM1 in the central nervous system.^95^ Higher levels of cNCAM identified in patient CSF^92–94^ may reflect reduced polysialylation of the 180 kDa NCAM isoform in brain, consistent with measures of reduced PSA-NCAM expression in postmortem schizophrenia hippocampus, DLPFC, and mPFC.^88,89^ Interestingly, CSF cNCAM levels of patients in very early stages of schizophrenia also correlate with ventricular volume increases that occur during the 2-year period following first-episode psychosis.^95^

In addition to protein-level evidence suggesting that defective NCAM polysialylation is associated with the pathogenesis of schizophrenia, genetic studies further support this. Polysialylation of NCAM1 is carried out by two polysialyltransferase enzymes: ST8SIA2 (also called STX, ST8SiaII, or SIAT8B) and ST8SIA4 (also called PST, ST8SiaIV, or SIAT8D).^84,96^ Evidence from patients in several geographic populations (Australia, Canada, China, Japan, and Spain) have implicated the chromosomal region 15q26 or the ST8SIA2 gene as a genetic risk factor for neuropsychiatric disorders, such as schizophrenia and bipolar disorder.^97–103^ Schizophrenia-associated SNPs of ST8SIA2 expressed in model systems yield a gene product with impaired function and reduced polysialylation.^97,104^

Another glycan structure, core fucose, has also been reported to be reduced in schizophrenia.^54^ Core fucose is produced by the activity of α-1,6-fucosyltransferase 8 (FUT8), and FUT8 protein levels are reduced in schizophrenia.^54^ Interestingly, core fucosylation of *N-*glycans appears to be a prerequisite of PSA attachment.^105^ Thus, reduced FUT8 expression may also contribute to lower levels of PSA-NCAM in schizophrenia. Model systems with impaired ST8SIA2 or FUT8 expression exhibit phenotypic features of schizophrenia.^56,104,106–108^ Models of impaired FUT8 expression also demonstrate AMPA-associated molecular and behavioral phenotypes similar to schizophrenia,^56,108^ which suggests that reduced core fucosylation of one or more AMPA receptor subunit *N-*glycans may contribute to some pathophysiological features of schizophrenia. Another sialyltransferase that attaches an α-2,6-Neu5Ac residue to terminal Gal residues demonstrates increased activity in samples of schizophrenia patient plasma.^109^

## Evidence for altered *N*-myristoylation in schizophrenia

*N*-myristoylation occurs co-translationally on the N-terminal glycine (Gly) of the Gly-Xaa-Ser/Thr/Cys consensus sequence (where Xaa is any amino acid), following the removal of the initiating methionine (Met).^15,16,21^ Many *N*-myristoylated proteins are involved in intracellular signaling pathways or have functions that require association with a specific organelle.^15,20,21,110,111^ *N*-myristoylation exerts a steric force to ensure the proper spatial orientation of molecules for correct interactions at or between membranes.^110,111^

A single *N*-myristoyl PTM is often insufficient for secure membrane attachment or cell membrane targeting without the addition of a second lipid, most often a palmitoyl group, or the presence of a polybasic domain within the core protein structure.^16,20^ The presence of a polybasic domain is a common feature of *N*-myristoylated substrates and the combination of *N*-myristoylation and phosphorylation acts as an electrostatic switch to influence the strength of the protein–membrane interaction.^21^ The electrostatic switch modulates intracellular signaling pathways by regulating protein–protein interactions between associated signaling molecules as well as by altering the electrochemical properties of the local membrane.

**Fig. 5:**
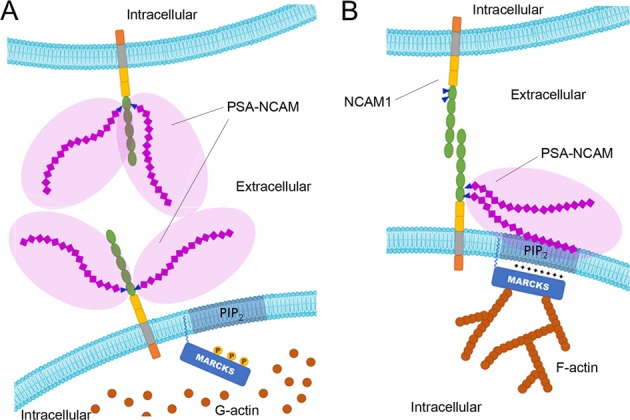
PSA-NCAM and *N*-myristoylated MARCKS. Schematic of the interaction between *N*-myristoylated MARCKS and PSA-NCAM. **a** Phosphorylation of MARCKS reduces the association between the polybasic domain of *N*-myristoylated MARCKS and PIP_2_-enriched membrane domains. PSA-chains on NCAM1 expressed on apposing cellular membranes produces steric hindrance due to the strong negative charge of PSA (indicated by shaded region) and inhibits the direct interaction of NCAM1 molecules necessary for synaptogenesis and/or synapse strengthening between neighboring cells. **b** Non-phosphorylated MARCKS polybasic domains can associate with PIP_2_-enriched membrane domains. Interactions within the lipid bilayer between intracellular MARCKS and extracellular PSA-NCAM serve as an electrostatic switch that facilitates MARCKS role in cross-linking actin filaments and permits changes to the cytoskeleton that alter cell morphology. When PSA chains interact with MARCKS, terminal IgG domains of PSA-NCAM are no longer prevented from interacting with NCAM1 on apposing cell membranes (shown without PSA modification) and synaptogenesis or synaptic strengthening can occur.

Evidence for dysregulated *N*-myristoylation in schizophrenia is currently limited to findings related to the defective function, localization, or expression of key substrates of this modification, such as the protein MARCKS. In schizophrenia there is reduced expression of MARCKS and phosphorylated MARCKS (pMARCKS).^112^ This is particularly interesting in the context of PTM-associated abnormalities in schizophrenia because MARCKS and PSA-NCAM interact via apposing sides of the plasma membrane to regulate actin cytoskeletal dynamics (Fig. 5).^113^ The respective glycan and lipid PTMs on these proteins appear to be necessary for proper interaction of their substrates. The antiadhesive properties of large, flexible, negatively charged PSA chains on NCAM facilitate structural changes associated with synaptogenesis, axon migration, and neurite outgrowth, and serve as an important regulator of spatiotemporal aspects of neuroplasticity during critical neurodevelopmental periods and throughout life.^86,114^ Polysialylation of NCAM on the extracellular side of the membrane influences these processes via its interaction with MARCKS located on the cytosolic side of the cell membrane.^113^ Myristoylated, membrane-associated MARCKS cross-links filamentous (F) actin with the cell membrane and facilitates structural adaptations in response to cellular stimuli.^115^ F-actin preferentially binds to non-pMARCKS when the *N*-myristoyl PTM acts as a membrane tether and the non-phosphorylated polybasic domain closely associates with the cell membrane.^115^ This allows the negatively charged PSA chains on the extracellular portion of transmembrane NCAM to interact with the positively charged polybasic domain of intracellular MARCKS within the lipid bilayer of the plasma membrane. The interaction alters the electrochemical properties of the local membrane microdomain to permit dendritic spine elongation, synaptogenesis, and cell motility.^113^ In schizophrenia there is evidence of reduced total and pMARCKS,^112^ reduced polysialylation of NCAM,^88,89,95^ as well as reduced F-actin, increased globular (G) actin, and a reduced F-/G-actin ratio.^116^ Taken together, abnormal PTM status of either or both NCAM and MARCKS may alter the spatial dynamics of the PSA–MARCKS interaction and thereby contribute to abnormal dendritic spine morphology in this illness.

## Evidence for altered prenylation in schizophrenia

In contrast to N-terminal modifying *N*-myristoylation, prenylation (also called isoprenylation or polyisoprenylation) is the enzymatic addition of an isoprenoid group, either farnesyl or geranylgeranyl, to C-terminal Cys within a particular consensus sequence.^15^ Prenylated proteins are only synthesized in eukaryotic cells and represent ~2% of mammalian proteins.^14^ Members of the Ras, Rac, Rho, and Rheb protein families are some of the main substrates of prenylation,^14,15,21,117^ although prenylation is not exclusive to these small GTPases. Prenylation targets proteins to the cellular plasma membrane and/or endomembranes, such as those which define intracellular organelles. Given the affinity of this lipid modification for endomembranes, prenylated proteins often require a second hydrophobic motif for targeting to the plasma membrane. This second motif may be an additional lipid modification, such as *S*-palmitoylation, or the presence of a positively charged polybasic domain, such as a Lys-rich region, within the substrate amino acid sequence. Similar to the *N*-myristoyl electrostatic switch, polybasic domain-containing prenylprotein affinity for plasma membranes can be modulated by phosphorylation of Ser/Thr residues within the domain, which reduces the prenylprotein–membrane interaction.^15,21^ Prenylation can also directly or indirectly influence protein–protein interactions by mediating conformational changes, introducing steric hindrance, or influencing protein spatiotemporal distribution in a substrate-specific manner.^14,21^

**Fig. 6:**
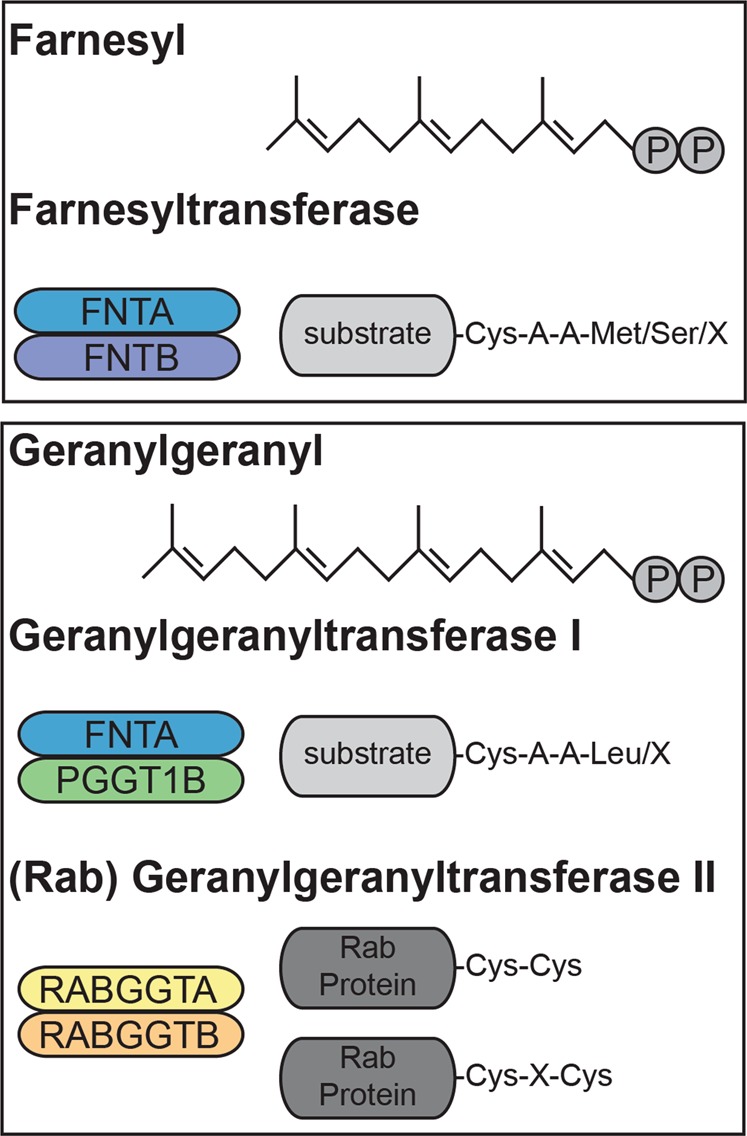
Prenyltransferase enzymes. Diagram showing chemical structures of the farnesyl and geranylgeranyl lipid donor molecules; and schematics of α and β subunit pairs that make up functional FT, GGT-1, and RGGT prenyltransferase enzymes and substrate molecules bearing the preferred consensus sequence for the corresponding prenyltransferase. In the consensus sequences shown, “A” represents any aliphatic amino acid and “X” represents any amino acid. Although FT and GGT-1 demonstrate preference for consensus sequences that terminate with Met, Ser, or Leu; they can also prenylate substrates with alternate residues in the “X” position of the CAAX consensus sequence. RGGT often transfers two geranylgeranyl groups, one on each C-terminal Cys residue, onto Rab protein substrates.

Bioinformatic assessment of multiple genetic datasets has identified differential gene expression of prenylation-associated enzymes in schizophrenia that varies by brain region and cortical layer.^118^ Three enzyme complexes catalyze protein prenylation: farnesyltransferase (FT), geranylgeranyltransferase I (GGT-1), and Rab geranylgeranyltransferase (RGGT; also called geranylgeranyltransferase II). Each of these is composed of an α and β subunit; FT and GGT-1 have the same α subunit and different β subunits, while RGGT subunits are not present in the other prenyltransferases^14,15,21,117^ (Fig. 6). Consequently, RGGT only mediates the geranylgeranylation of Rab family proteins, while FT and GGT-1 are able to prenylate a variety of substrates. Investigation of the protein expression levels of prenyltransferase subunits revealed reduced expression of FNTA, PGGT1B, and RABGGTA in schizophrenia DLPFC.^118^ This suggests that all forms of prenylation—farnesylation, typical geranylgeranylation, and Rab-specific geranylgeranylation—may be impaired in schizophrenia. Given that the expression of both α and β subunits of GGT-1 are reduced, substrates of typical geranylgeranylation are likely to be the most impacted by altered prenylation in schizophrenia.

## Evidence of abnormal *S*-Palmitoylation schizophrenia

Many proteins require dual lipid PTMs to ensure secure interactions with the plasma membrane. *S*-palmitoylation on Cys residues of prenylated or *N*-myristoylated proteins often provides the additional hydrophobic interaction necessary for stable membrane associations.^16,20,21^ Although *S*-palmitoylation often occurs with prenylation or *N*-myristoylation, the presence of other lipid PTMs is not a prerequisite for attachment of the palmitoyl group. The specific functional effects of *S*-palmitoylation are substrate dependent, and *S*-palmitoylation status of key proteins can result in diverse effects on many aspects of cellular function and neurotransmission both in tandem with or independent of other PTMs.^21,119–125^

In schizophrenia DLPFC, the total level of S-palmitoylated protein expression is reduced by 20% compared to non-psychiatrically ill subjects.^126^ In addition to finding reduced total S-palmitoylation, the palmitoylation status of some known substrates was measured. vesicular glutamate transporter 1 (VGLUT1), myelin basic protein (MBP), and Ras exhibited lower levels of palmitoylation when assessed by acyl-biotin exchange assays. Decreased S-palmitoylation of these proteins could play a role in myelination and white matter abnormalities reported in schizophrenia^127,128^ Interestingly, this study did not detect altered protein expression of a subset of enzymes that attach or cleave palmitoyl groups in schizophrenia. However, given that several proteins, including Ras family proteins, are prenylated prior to being palmitoylated, altered prenyltransferase expression and defective prenylation of some molecules may contribute to reductions of S-palmitoylation in schizophrenia.

Unlike other forms of lipidation, *S*-palmitoylation is reversible and cycles of de-/re-palmitoylation can have wide-spread pleiotropic effects depending on the affected substrates.^14,15^ Dynamic cycles of de- and re-palmitoylation are known to promote protein shuttling between subcellular compartments, and can introduce tertiary structural features that influence substrate function or activity. A unique class of lipid transferase enzymes catalyzes the attachment of palmitate to Cys. These protein acyltransferases (PATs) contain an Asp-His-His-Cys (DHHC; aspartate-histidine-histidine-cysteine) sequence within a Cys-rich domain and, because of the shared amino acid sequence, are typically referred to as DHHC proteins.^129^ Acyl-protein thioesterases (APTs) and palmitoyl protein thioesterases (PPTs) catalyze the removal of palmitoyl groups. The dynamic interplay between PATs and APTs/PPTs on substrate proteins is an essential element in determining the subcellular localization and movement of palmitoylproteins within the cell. In the context of synaptogenesis and synapse maintenance arising from long-term potentiation or long-term depression (LTP or LTD, respectively), the *S*-palmitoylation state of key proteins influences the clustering and stability of neurotransmitter receptors at the synapse, thereby impacting the firing rate and/or amplitude of action potentials.^119–121,123–125^ A recent study compared the enzyme activity of PPT1 in blood serum from first episode psychosis schizophrenia patients and healthy comparison subjects and found that increased PPT1 activity in patients was positively associated with higher Positive and Negative Syndrome Scale (PANSS) scores.^130^ Increased activity of PPT1, despite the lack of evidence for altered protein levels the enzyme, could also explain reduced total S-palmitoylation identified in postmortem patient brain and indicates that defects of this PTM contribute to behavioral phenotypes of schizophrenia.

Impaired protein S-palmitoylation in schizophrenia is also consistent with DHHC protein alterations in the disorder. Genes encoding ZDHHC18 and ZDHHC5 were recently found to be significantly associated with schizophrenia risk,^131^ and ZDHHC5 was identified in a recent GWAS study reporting 108 schizophrenia-associated loci.^132^ The gene encoding ZDHHC8 is located on the affected chromosomal region in 22q11 deletion syndrome and estimates show that ~30% of patients with 22q11 deletion syndrome will develop schizophrenia or psychotic symptoms.^133–137^ Abnormalities of ZDHHC8 expression have been implicated in molecular defects which are common in schizophrenia; however, genetic associations between ZDHHC8 and schizophrenia risk have been contradictory.^131,133–146^

## Substrate-specific lipidation alterations in schizophrenia

Hh family proteins are the substrates of two unique lipid modifications: *N*-palmitoylation and cholesterylation. Hh proteins are known to be essential for proper (neuro)development and mutations which affect their lipidation can result in severe developmental defects and possible embryonic lethality.^17,18,147,148^ Following translation, mammalian Hh proteins are modified by Hh acyltransferase (Hhat), which attaches a palmitoyl group to the N-terminal Cys.^15,17,148^ This form of palmitoylation, called *N*-palmitoylation, is different from *S*-palmitoylation because the attachment is via a stable amide bond as opposed to the more labile thioester bond of *S*-palmitoylation.^15,17,148^ In a Bulgarian population, a SNP in the Hhat gene (rs7527939) is highly correlated with schizophrenia susceptibility, and has been suggested that this may lead to altered cell migration and neurodevelopment in the disorder.^149^ Another rare lipid PTM, cholesterylation of Hh, occurs via the C-terminal autoprocessing domain which results in the incorporation of cholesterol and release of the C-terminal autoprocessing domain. Although *N*-palmitoylation and cholesterylation occur independently, both play important roles in the function and signaling properties of Hh proteins. Dual lipidation is important for both membrane tethering of Hh proteins and also for their interaction with receptors on both the signaling cell membrane and the receiving cell membrane. Some antipsychotic and antidepressant medications (clozapine, chlorpromazine, haloperidol, and imipramine) have been shown to regulate Hh signaling by modulation of 7-dehydrocholesterol reductase, and abnormalities of cholesterol-associated metabolic pathways are evident both in schizophrenia and in response to antipsychotic treatment.^150–153^ Together, these reports suggest that Hh family protein lipidation abnormalities may play a role in some features of the disorder and/or the efficacy of certain antipsychotic medications.

Another unique form of protein lipidation, octanoylation of ghrelin, might contribute to metabolic abnormalities associated with antipsychotic administration. Ghrelin is activated by octanoylation, the addition of octanoate via an oxyester bond on Ser3.^15^ Ghrelin activation regulates cellular glucose homeostasis and metabolic pathway activity, and is the only known orexigenic (appetite stimulating) hormone.^19^ Ghrelin-mediated signaling pathways are of interest in schizophrenia due to the metabolic side effects of many antipsychotic medications.^154^ Serum levels of total ghrelin have been reported to be significantly reduced in patients with schizophrenia following treatment with either olanzapine or clozapine when compared to non-psychiatrically ill comparison subjects.^155,156^ Reduced ghrelin is also reported following treatment with olanzapine when compared to a baseline measure obtained prior to antipsychotic administration.^157^ Only one study has assessed the level of octanoylated (active) ghrelin in patients. Interestingly, the level of octanoylated ghrelin in patient serum did not change over a 16-week course of olanzapine administration in the face of reduced total ghrelin at 8-, 12-, and 16-weeks of olanzapine treatment.^157^ This indicates that the relative amount of active versus inactive (non-octanoylated) ghrelin is increased with antipsychotic treatment, and suggests a potential mechanism contributing to antipsychotic-induced metabolic dysregulation in schizophrenia.

## Future directions

From a translational perspective, glycobiology has yielded promising advances in clinical diagnosis, evaluation of medication response, and identification of pharmacologic targets for the treatment of multiple medical illnesses.^158–160^ The branching nature and variable position of glycosidic bonds contributes to structural diversity of glycoproteins and can provide uniquely targetable substrates for therapeutic intervention.^12,161^ Advances in the sensitivity of technologies used to assess protein PTMs have also become available, and more comprehensive glycomic and lipidomic analyses of patient samples are possible with improvements in mass spectrometry methods and tools. Improvements in metabolite and enzyme functional analyses can aid in the identification of abnormal lipid species and glycan structures.^14,158,159,162^ Although model systems are useful tools in the research armamentarium, care must be taken in interpreting findings from immortalized cell lines and vertebrate models due to species-specific patterns of glycosylation and lipidation, as well as known perturbations of these processes in cancer biology. Induced pluripotent stem cells derived from patient samples could offer a more useful ‘model’ to investigate PTM dysregulation in schizophrenia, yet again the caveat that PTM pathways are highly involved in many aspects of cell and molecular developmental processes needs to be kept in consideration. Given that some alterations of brain protein glycosylation and lipidation are reflected in the pattern of biomolecules and metabolites expressed in peripheral body fluids,^88,89,95,159,160^ research elaborating the pathological relevance of specific glycoproteins, lipoproteins, or PTM-associated enzymes may yield promising non-invasive methods of monitoring patient response to treatment. Future assessments linking findings in postmortem brain with biomolecule expression patterns in patient peripheral fluids could also provide novel targets for therapeutic or diagnostic biomarker discovery.

Multiple lines of evidence support the hypothesis that dysregulation of PTM processing mechanisms and consequent protein PTM status abnormalities play an important role in the molecular pathophysiology of schizophrenia (Tables 1–3). Given the essential functions mediated by adornment of proteins by glycan and lipid PTMs, it is likely that dysfunction of these processes is a feature of schizophrenia cellular biology. A variety of methods have been used to identify disruptions to PTM-processing pathways and to determine substrate PTM status in schizophrenia, but the body of literature exploring these pathways and effects on substrate activity remains relatively sparse. As technologies advance and assessments of glycosylated and/or lipidated protein isoforms are performed, inconsistences or contradictions which have arisen from prior reports in schizophrenia may be reconciled. Previously promising research directions which have been stalled or abandoned due to inconsistencies between genetic and proteomic assessments might be reconsidered with a fresh perspective on the importance of glycan and lipid attachment downstream of transcription and translation.
